# Xen45 gel stent ab interno trimming for ostium occlusion: case report

**DOI:** 10.1186/s12886-021-02207-8

**Published:** 2021-12-27

**Authors:** Filippo Tatti, Pietro Gentile, Lorenzo Mangoni, Giuseppe Demarinis, Pietro Napoli, Maurizio Fossarello

**Affiliations:** Department of Surgical Sciences, Eye Clinic, University of Cagliari, Via Ospedale 48, 09124 Cagliari, Italy

**Keywords:** Glaucoma, MIGS, XEN, Complications, Case report

## Abstract

**Background:**

Despite the XEN gel stent low-risk profile, various complications following the implant have been described, including internal and external occlusion, with a consequent postoperative rise in intraocular pressure (IOP). In this case report we aimed to present a XEN45 stent internal occlusion successfully treated by trimming in situ its proximal end with a 25 G vitreous scissors using a bimanual technique.

**Case presentation:**

A 63-year-old male patient affected by primary open angle glaucoma (IOP = 25 mmHg) and a full thickness macular hole in his right eye, underwent ab-interno Xen gel stent implantation and, 1 month later, a 25 G vitrectomy surgery. Despite a significant IOP reduction after stent implant, 6 days after vitrectomy, IOP increased (25 mmHg) and the conjunctival bleb flattened following occlusion of stent internal ostium by a clot of presumed fibrinous material. The Nd:YAG laser failed to remove the clot, so that we decided to snip a small bit of the proximal end of the Xen tube (about 0,5 mm length) with a 25 G vitreous scissors, using a bimanual technique. In the postoperative day 1 and month 1, the IOP was 8 mmHg and 12 mmHg, respectively. The Anterior Segment OCT confirmed a functional, layered, filtering bleb, and the normal appearance and patency of the XEN proximal segment. No side effects from the intervention were observed.

**Conclusions:**

Ab interno trimming with vitreous scissors of the occluded proximal end of the XEN stent may represent a safe, rapid and efficient method to restore aqueous humor subconjunctival drainage.

## Background

Glaucoma is the leading cause of irreversible vision loss worldwide. In 2013, the number of affected people was estimated to be 64.3 million, increasing to 111.8 million in 2040 [1].

The only modifiable risk factor is the intraocular pressure (IOP); hence its lowering is considered the therapeutic approach for glaucoma [2]. This goal can be performed by medications, usually preferred as a first-line therapy, laser techniques and surgery [3]. Trabeculectomy remains the standard surgical procedure, although it may lead to significant complications [4]. Although it was reported a higher rate of complications in trabeculectomy versus the mini glaucoma device implantation, some device–related late complications due to shunt dislocation or extrusion have been described [5]. Minimally-invasive glaucoma surgeries (MIGS) have been developed to provide safer and less traumatic surgical interventions, aiming at curtailing the need for topical medications [6].

The Xen45 (Allergan Plc, Dublin, Ireland) is a tubular implant 6-mm-long and with an inner lumen of 45-μm, made of hydrophilic collagen, comprised of cross-linked porcine gelatin, which drains aqueous from the anterior chamber (AC) to the subconjunctival space (SS), bypassing the resistance of the dysfunctional trabecular meshwork [7]. The implant is injected ab interno through a clear corneal incision with a pre-loaded, single-use injector with a 27-gauge needle. The ideal stent placement should leave 2.0 mm of exposed implant in the subconjunctival space (preferentially in a more superficial layer than the sub-Tenon space), 1.0 mm in the AC, and 3.0 mm tunneled through sclera. The lumen size and length of the implant are designed to provide approximately 8 mm pressure resistance according to the Hagen-Poiseuille equation, so as to confer protection against hypotony [7].

Anterior segment optical coherence tomography (AS-OCT) enables tomographic imaging not only of the filtering bleb, but also the visualization of the implant itself, its subconjunctival and intrascleral pathway, and even the intracameral segment [8].

Several studies demonstrated the XEN gel stent efficacy in lowering IOP [9, 10].However, despite its low-risk profile, various complications following Xen implant have been described, including internal and external occlusion, with a consequent postoperative rise in IOP [9, 10].

There are several methods of unblocking the end of glaucoma stents occluded by fibrin clots such as Nd:YAG laser lysis [11],removal by ILM forceps [12], and Intracameral tissue plasminogen activator [13].Although the accidental or intentional trimming of the Xen stent external ostium has been previously described [14–16], the ab interno cutting of tube end has been described only in eyes with Baerveldt GDD implantation [17].

In this report we describe a case of Xen internal ostium obstruction by presumed fibrinous material, which occurred after vitreoretinal surgery, and its management.

## Case presentation

### History

A 63-year-old man was referred to our clinic for primary open glaucoma in both eyes, with an intraocular pressure of 25 mmHg in the right eye despite topic medications (Dorzolamide, Timolol, Travoprost). At the initial visit the anterior segment of the pseudophakic right eye was normal and the best corrected visual acuity was 20/200. The fundus examination showed peripapillary atrophy, glaucomatous neuroretinal rim loss (cup/disk ratio 0,7) and a full thickness macular hole in his right eye. The patient underwent an ab-interno gel stent implantation in the upper nasal quadrant (Xen45, Allergan) after a subconjunctival injection of mitomycin-C (0,1 ml 0,02%). After hydrating the incisions, 0.1 ml of 1% cefuroxime was injected in the anterior chamber and 4 mg/1 ml of dexamethasone phosphate subtenon (Decadron®, Farmaceutici Caber SpA, Italy). Post-surgery care included antibiotic prophylaxis and topical corticoids in decreasing dosage during 1 month. The IOP was 6 mmHg and 10 mmHg in post-operative day 1 and post-operative day 15, respectively. One month after the implantation, the patient underwent retinal surgery in his right eye. The macular hole was successfully closed with a 25-G pars plana vitrectomy (Constellation vitrectomy machine, Alcon Laboratories, Inc., Fort Worth, TX, USA), using the inverted ILM flap technique [18].

The surgery was uneventful and subconjunctival dexamethasone was used to minimize inflammation. Topical medication with combined antibiotic and steroid drops was prescribed for 1 week.

**Fig. 1:**
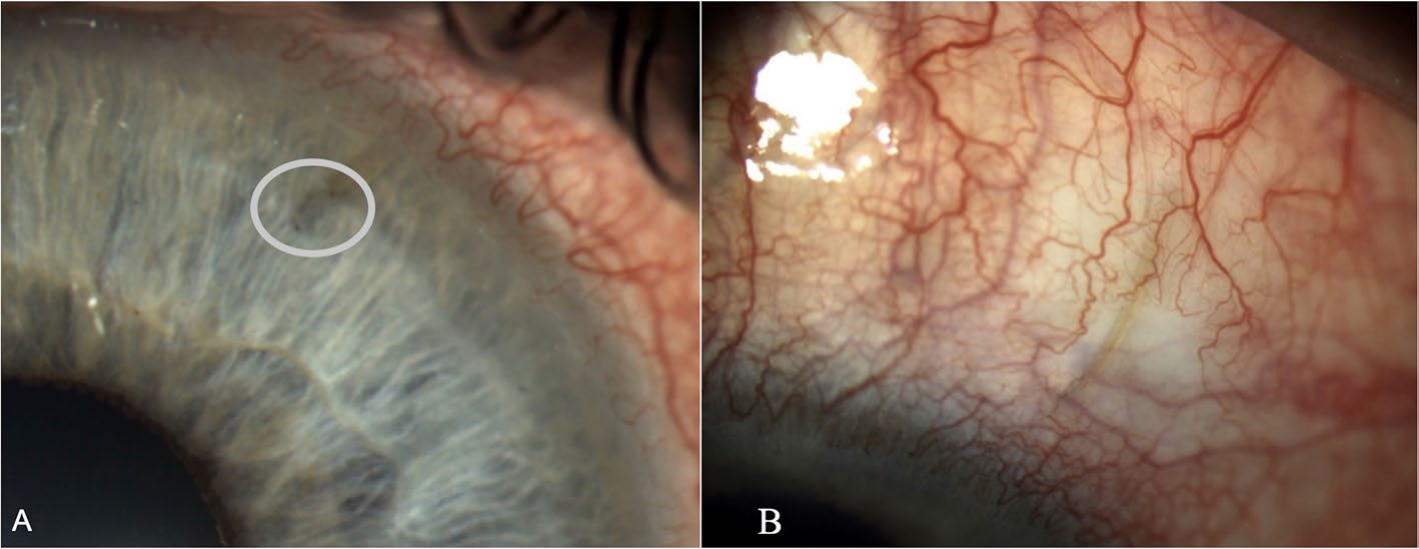
Biomicroscopy image showing: **A** The Xen45 anterior chamber portion (about 1 mm) obstructed by a translucent clot of fibrinous material surrounding the internal end (white circle); **B** A segment of the Xen45 is visible under a flat conjunctival bleb

**Fig. 2:**
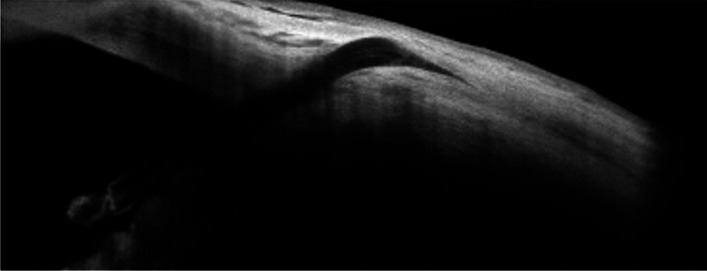
The AS-OCT shows the fibrinous material surrounding the inner end and occluding the lumen of the Xen45; a non-filtering, non-functional, flat conjunctival bleb is also visible

Six days after vitreous surgery the IOP spiked to 25 mmHg, and the anterior segment examination (Takagi TD10 Eye Cam on a Takagi 700GL Slit lamp) showed a flattening of the conjunctival bleb and a translucent clot covering the internal ostium of the XEN. (Fig. 1) At the AS-OCT (AngioVue®, Optovue, Fremont, CA, USA) the conjunctival bleb appeared flat and non-functional, and the clot stood out as hyperreflective material which we presumed to be fibrin, occluding the internal end of the stent (Fig. 2).

Lysis of the fibrin clot was attempted with YAG-laser (1,1 mJ of power), but the tube remained occluded and the IOP elevated. Therefore, an ab interno revision of the gel stent was performed.

### Surgical procedure

A surgical procedure was performed according to the following steps after obtaining written informed consent from the patient.

**Fig. 3:**
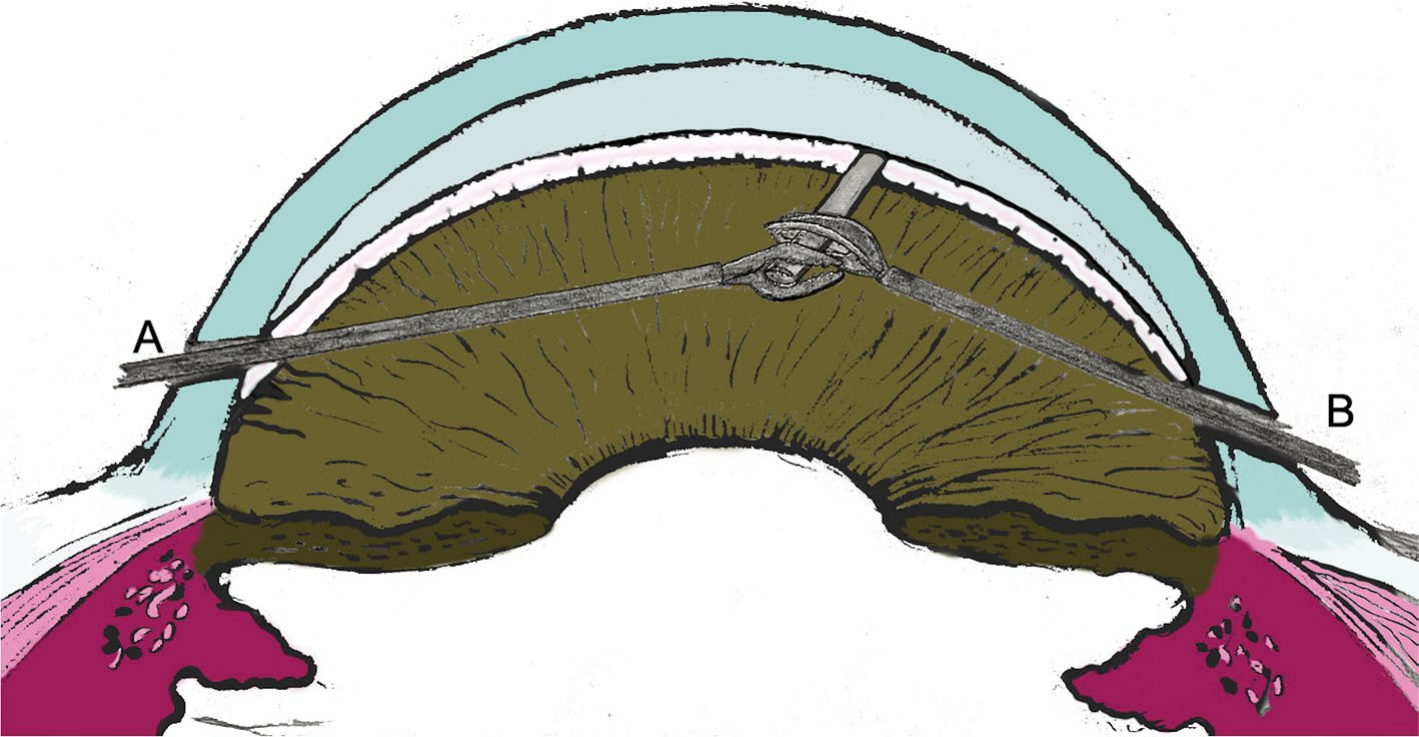
Digital reproduction of the two-handed technique, with a 25-gauge inner limiting membrane (ILM) forceps (**A**) and a 25 G straight vitreous scissors (**B**), used in order to both immobilize and truncate the end of the stent

- After application of topical anesthesia with the usual sterile conditions in the operating room, one paracentesis site was created at 90° with respect to the tube (approx. at 10 o’clock) by using a 15° Knife (Stab 15° Safety Knife, Surgistar, California)
- Trypan blue (Vision Blue, DORC international, BV Zuidland, Netherlands) was injected into the anterior chamber to stain the clot and to verify the extension of tube occlusion.
- Viscoelastic was introduced in the anterior chamber and a careful fibrin clot removal was attempted with a 25-gauge inner limiting membrane (ILM) forceps (Revolution DSP 25+ Serrated Forceps, Alcon-Grieshaber, Fribourg, Switzerland); however, the clot was strongly adherent to the tube end, and it did not clear.
- A second paracentesis site was created at 90° with respect to the Xen tube position (approx.at 4 o’clock), and a 25 G straight vitreous scissors (Revolution DSP 25G Curved Scissors, Alcon-Grieshaber, Fribourg, Switzerland) was inserted to snip the proximal end of the Xen tube (a small segment of about 0,5 mm length), flush to the ILM forceps grabbing distally the tube. The flexible nature of the stent required a two-handed technique in order to both immobilize and truncate the end of the stent. (Fig. 3)
- The viscoelastic and the excised tube fragment were then removed, and the AC was irrigated with balanced salt solution (BSS) to induce subconjunctival bleb formation. The drainage efficiency was further verified by trypan blue injection into the AC.
- Finally, the corneal incisions were closed by hydrosuture.

**Fig. 4:**
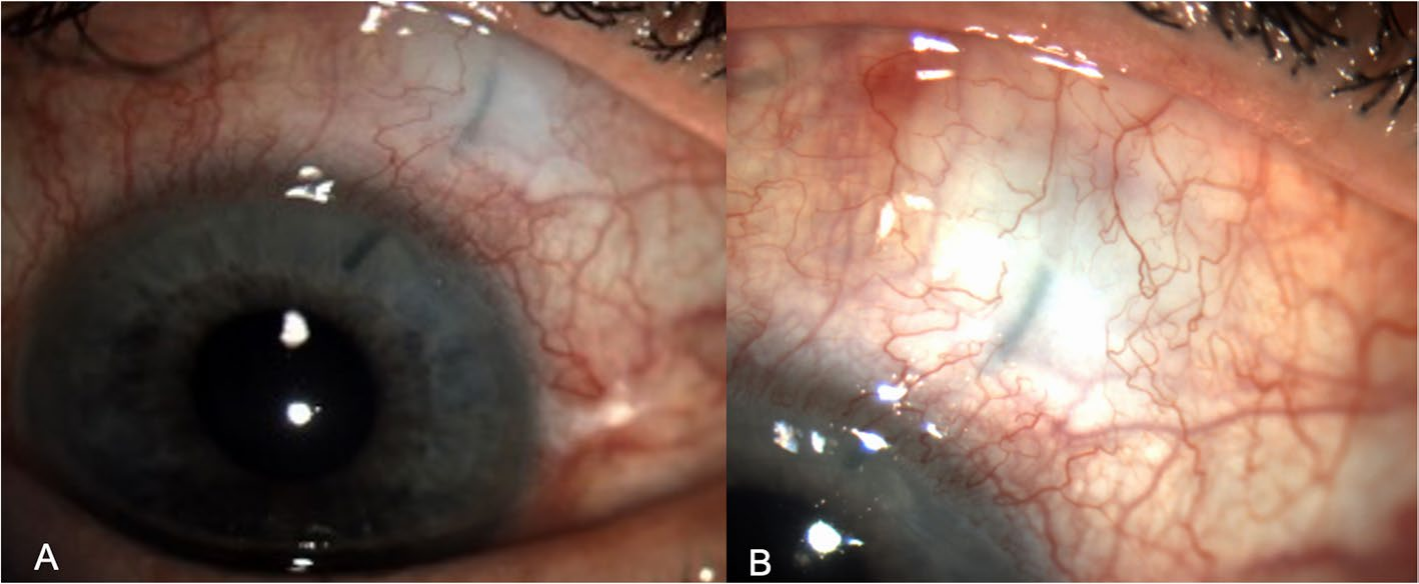
Anterior segment image acquired on postoperative day1 shows the XEN gel stent visible in the anterior chamber (**A**) and an open diffuse filtering bleb (**B**). The tube is stained with blue, confirming the open drainage pathway from the anterior chamber.

**Fig. 5.**
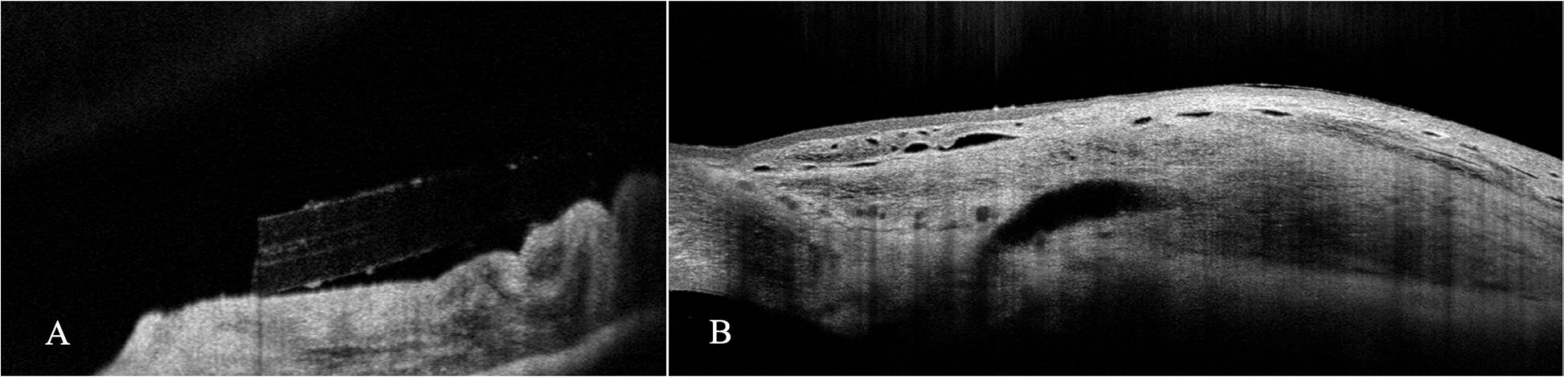
AS-OCT image evidences the patency of the internal ostium of the tube (**A**) and a functional, layered, filtering conjunctival bleb (**B**)

In the postoperative day 1, the IOP was 8 mmHg; the anterior segment examination showed an open diffuse filtering bleb, a normal appearing new internal ostium, and a sustained blue staining of the stent [19]. (Fig. 4) The AS-OCT confirmed a functional, layered, filtering bleb, and the normal appearance and patency of the stent. (Fig. 5) No side effects from the intervention were observed.

Topical medication with combined antibiotic and steroid drops was prescribed for 2 weeks. One month after surgery the patient showed IOP =12 mmHg, a visual acuity of 20/40, a patent internal ostium, and an open diffuse filtering bleb.

## Discussion and conclusions

Despite several studies have demonstrated the effectiveness and safety of the Xen45, a variety of postoperative complications have been reported [9, 10].In particular, early lumen occlusion can occur in 4% of Xen stent procedures for primary open-angle glaucoma (blood, fibrin, air bubble or iris) [20], or later due to continued inflammation [21].

Vitreoretinal surgery is considered a possible cause of AC fibrin formation [22]: in our patient a presumed fibrin clot formed at the internal end of the stent 1 week after macular hole surgery.

Different methods of unblocking the proximal end of glaucoma stents occluded by fibrin/blood clots have been described: Nd:YAG laser lysis [11], removal by ILM forceps [12], and Intracameral tissue plasminogen activator [13].

Despite the Nd:YAG laser was effective in a case of XEN occlusion, in our case it was unsuccessful. One-hand removal by ILM forceps was described to be successful in a case of blood clot internal occlusion [12], but was ineffective in our case, probably due to the high adhesiveness of the fibrin clot to the tube end. Intracameral tissue plasminogen activator has been used successfully in eyes with valved glaucoma drainage implants to clear or prevent tube occlusion by fibrin/blood clots, but its use has never been reported in eyes with Xen stent blockage.

Although unintentional Xen stent truncation of external end has been observed during needling procedure for in-growing fibrotic tissue [14–16], and intentional truncation has been performed with YAG laser in a case of threatened conjunctival extrusion [23], ab interno trimming of tube end has been described only in eyes with Baerveldt GDD implantation [24].

Indeed, doubts and concerns may arise from manipulation and surgical trimming, with consequent shortage, of the stent tube.

Undoubtedly, even cautious clamping of the internal end may retract the stent into the anterior chamber, and attention must be paid to limit its sliding. Shortage of the tube in the range of 1 mm seems to be well tolerated: In our patient, the IOP dropped to 8 mmHg 1 day after trimming, to stabilize at 12 mmHg after 1 month. Similarly, in reported cases of Xen external amputation of the subconjunctival portion, the shortage length ranged from 0.7 to 1,1 mm, and did not seem to impair the expected normal resistance (set at 8 mmHg) of the draining device [14–16].

As previously reported, the XEN 45 implant was designed to limit secondary hypotony by virtue of its length and width according to the Hagen-Poiseuille equation [7], Resistance to flow is directly proportional to the length and inversely proportional to the radius of the tube to the fourth power [24]. Thus, a shorter tube provides a smaller pressure differential, or less resistance to flow. Contrarily, Jansonius postulated that there is no physical basis for a relationship between tube length and intraocular pressure after glaucoma drainage implant surgery [25]. Considering this aspect was referred to glaucoma tubes, further studies are needed to evaluate the applicability of Hagen-Poiseuille law to Xen gel stent.

However, once the bleb has matured and subconjunctival fibrosis has occurred, the resultant IOP will ultimately be limited by subconjunctival resistance [24].

Further study will be needed to determine the long-term effects of shorter Xen45 tube on IOP control and wound healing response of the bleb.

In conclusion, to our knowledge, this is the first case reporting ab interno trimming of the proximal end of Xen45 stent occluded by fibrinous material. The post-operative course and IOP decrease were satisfactory, and the AS-OCT imaging confirmed the bleb had restored, and the tube shunt had completely cleared of obstruction. Our bimanual method appears to be minimally invasive, rapid and efficient to reestablish the drainage flow, and may be easily applied to similar cases, when other methods have failed.

## Data Availability

The datasets used and analyzed during the current study are available from the corresponding author on reasonable request.

## Abbreviations

AC: Anterior Chamber
AS-OCT: Anterior Segment – Optical Coherence Tomography
BSS: Balanced Salt Solution
ILM: Inner Limiting Membrane
IOP: Intraocular Pressure
MIGS: Minimally-invasive glaucoma surgeries
SS: Subconjunctival Space
